# A cross-sectional study investigating impressions and opinions of medical rehabilitation professionals on the effectiveness of the Ponseti method for treatment of clubfoot in Harare, Zimbabwe

**DOI:** 10.1186/s40945-016-0021-5

**Published:** 2016-06-30

**Authors:** N. Munambah, M. Chiwaridzo, T. Mapingure

**Affiliations:** 1 Department of Rehabilitation, University of Zimbabwe, College of Health Sciences, P.O Box A178, Avondale, Harare Zimbabwe; 2 SANDI Rehabilitation, Lilongwe, Malawi

**Keywords:** Ponseti method, Clubfoot, Medical rehabilitation professionals, Harare, Zimbabwe

## Abstract

**Background:**

The Ponseti method of managing clubfoot was introduced in Zimbabwe in 2011. This followed massive training of health workers such as medical rehabilitation practitioners through a programme called the Zimbabwe Sustainable Clubfoot Programme. Today, the Ponseti method is the technique of choice for managing clubfoot in hospitals. However, since then, there is no published evidence documenting the efficacy and the relevance of the technique especially comparing to previously used methods. This is a significant shortcoming if sustainability issues are to be considered. Therefore, this study was designed to investigate the impressions and opinions of medical rehabilitation practitioners on the method in terms of its effectiveness, perceived challenges and possible recommendations for improvement of the technique application in their setting.

**Methods:**

A descriptive cross-sectional study was conducted targeting medical rehabilitation practitioners previously trained on the method and working in public or private clinics that offer clubfoot management in Harare. A questionnaire was self-administered to 41 participants who volunteered to participate in the study. Data from open-ended questions was analysed thematically. Statistica version 12 was used for analysis for quantitative data.

**Results:**

The Ponseti method was perceived as an effective method in the treatment of children with clubfoot by all the participants. All the participants 41 (100 %) felt that the method was relevant because of better clinical outcomes. Amongst challenges faced when using Ponseti method, 25 (61 %) participants agreed that caregivers to the children with clubfoot were not compliant to treatment. A total of 22 (54 %) participants felt that lack of adequate insight by the caregivers of this new method was a challenge which hinder progress in treating clubfoot.

**Conclusions:**

The medical rehabilitation professionals in Harare, Zimbabwe trained to use the Ponseti technique for the management of clubfoot, perceived the method as an effective method resulting in better clinical outcome than previous methods. This probably highlights the need to continue training medical rehabilitation professionals so that there is widespread use of the technique in the country. However, there is need to increase awareness of the method among caregivers to improve compliance, which is key to successful rehabilitation of the clubfoot.

## Background

Clubfoot is one of the most common anomaly in paediatrics orthopaedics, affecting approximately 1–2 per 1 000 live births worldwide [1–6]. The majority of the cases of clubfoot (80 %) have been reported to occur in developing countries [7]. In Zimbabwe, it is estimated that around 460 children are born with the clubfoot of various types each year [8]. A three-year retrospective study conducted at one large central hospital in Harare Zimbabwe reported a minimum incidence (MI) rate of 0.9 per 1 000 for congenital talipes equinovarus [9].

Congenital talipes equinovarus (CTEV) is the most common type of clubfoot, but there are other combinations possible such as calcaneovalgus, equinovalgus and calcaneovarus [10]. It is characterised by a complex, three-dimensional skeletal anomaly of the foot [6] with three underlying deformities which consists of a hind foot in equinus (planterflexion) and varus (inversion) combined with a cavus deformity (abnormally high arch) and an adductus component at the midfoot [10, 11]. If not treated, CTEV most often will result in permanent disability which can restrict a child to walk, play and perform activities of daily living such as self-care [12, 13]. Neglected clubfoot have been reported to cause disabilities that result in a lack of social integration for the individual affected, creating a long term psychological and financial burden for the family and community [7, 14]. Therefore, the goal of treatment is to obtain and maintain correction of the clubfoot so that a patient has a functional, pain free, plantagrade foot with good mobility and without calluses [15–17]. Treatment options have in the past included the Kite method, French taping method and surgery, the last being the treatment of choice for most orthopaedic surgeons [6, 7, 10, 14, 17].

Over the past decade, a new non-invasive treatment method known as the Ponseti method (PM) has been developed and has been gaining acceptance worldwide [5, 18]. The technique was first introduced around 1940s by Dr Ignatio Ponseti from the University of Iowa only to be repopularised worldwide a decade ago [19, 20]. It involves conservative techniques of manipulation, casting and a small percutaneous surgery to release the tendon of Achilles at the end [14, 21]. Studies have shown that when applied correctly it can achieve correction of the clubfoot deformity with excellent results [7, 22]. Open surgery has been reported to be avoided in 89 % of the cases by using the Ponseti technique of manipulation and casting [17]. A prospective follow up study conducted in India in 40 children with clubfoot treated by the PM for four years showed a significant difference between pre-treatment and the post-treatment Pirani scores (a measure of clubfoot severity) and goniometry values, indicating effectiveness of the technique in correcting clubfoot deformity [7].

In Africa, the technique has gained widespread acceptance in most countries as a treatment of choice for children with clubfoot [20, 23, 24]. In Uganda, the PM was started in 1999 but fully implemented in 2003 amidst challenges of lack of trained human resources, materials and financial support [20]. In South Africa, a study by Khan [23] indicated that the PM was first used in 2003 and has achieved high success rate in correcting the clubfoot deformity with substantial parental compliance. In Malawi, the PM has been evaluated and has been found to be suitable for treatment of uncomplicated cases of clubfoot even by non-medical personnel [24].

In Zimbabwe, the PM was introduced in 2011 by an organisation called Zimbabwe Sustainable Clubfoot Programme (ZSCP) to address the need for effective and sustainable treatment of children with clubfoot [8]. This need was identified through encountering cases of poorly managed clubfoot during community outreach and other research [8]. The ZSCP organised a series of training workshops for health care workers already involved in the management of clubfoot starting with the large central public hospitals in Zimbabwe [8]. The participants were awarded certificates of competencies after the training. Today, the PM is widely used in public and some private rehabilitation departments to manage children with clubfoot. Generally, treatment and management of clubfoot in Zimbabwe is usually done by medical rehabilitation practitioners (physiotherapists, occupational therapists and rehabilitation technicians) and medical doctors. The medical rehabilitation professionals administer all components of Ponseti treatment for the clubfoot except for the tenotomy. Additionally, the method is now taught in the local universities and schools to medical and rehabilitation (physiotherapists and occupational therapy) students as part of their curriculum to increase awareness of the technique.

However since its establishment, the evaluation of the PM has not been conducted in Zimbabwe. It is not clear if the technique is effective or relevant in the local context as compared to the previously used techniques such as the Kite method. There is no study documenting self-reported evidence evaluating the effectiveness of the PM against previously used methods. Therefore, this study sought to understand the general opinions and impressions of the primary health workers (medical rehabilitation practitioners) involved in the management of clubfoot using the PM about its effectiveness and relevance. The secondary aims were to highlight the challenges faced by these clinicians in using the PM and the possible strategies to improve its effectiveness. This evaluation is important for the trainers or funders (ZSCP) if sustainability issues of the training programmes are to be considered.

## Methods

### Study design and participants

This study was a cross sectional descriptive study with a quantitative design. The study was conducted in Harare, the capital city of Zimbabwe, targeting all medical rehabilitation centers from public hospitals to individual private clinics. There are three large public hospitals in Harare (Parirenyatwa Group of Hospital, Harare Central Hospital and Chitungwiza Central Hospital) all with well-established large rehabilitation departments. The ZSCP provides clinical and material support to these hospitals. Treatment of clubfoot is offered free of charge to the children in all the hospitals. In these hospitals, physiotherapists run the clubfoot clinics in collaboration with occupational therapists, rehabilitation technicians and hospital doctors who perform tenotomies under local anaesthesia.

To be included in this study, medical rehabilitation professionals had to be registered with the Medical Rehabilitation Practitioners Council of Zimbabwe (MRPCZ). The three group of professionals registered under MRPCZ with the clinical obligation to manage clubfoot patient in hospitals and clinics are physiotherapists, occupational therapists and rehabilitation technicians. Physiotherapists and occupational therapists are jointly trained for four years at the University of Zimbabwe, College of Health Sciences by the Department of Rehabilitation and graduate with an honours degree in either physiotherapy or occupational therapy. On the other hand, rehabilitation technicians are trained for two years at Marondera Rehabilitation Technicians Training School by physiotherapists and occupational therapists working under the Ministry of Health and Child Care for a diploma, partly covering aspects of physiotherapy, occupational and speech therapy. They are usually trained in large numbers so that they can be deployed as rehabilitation cadres to remotest areas including rural hospitals, provincial and district hospitals. In large central hospitals, rehabilitation technicians clinically work under the supervision of qualified physiotherapists and occupational therapists.

Additionally, only trained participants with a certificate of competence as proof were included in the study. Moreover, the trained rehabilitation professional should have treated at least two children with clubfoot using the PM after the training. Eligible participants not available in their clinical settings in the period of the survey were excluded from the study. At the time of the study, 51 medical rehabilitation practitioners were eligible, and 41 agreed and were available to participate in the study.

### Instrument

A researcher-developed questionnaire was used to collect data. The questions were derived from the literature. The questionnaire was divided into three sections. Section A collected socio-demographic data of respondents such as gender, age, profession, place of work (public or private) and years of clinical practice. Section B consisted of closed-ended questions asking respondents to indicate on preselected factors their impression or opinions for the PM. Open ended responses were also elicited in order for the respondents to freely give their opinions on the PM. Some of the questions asked whether the participants were using the PM in treating children with clubfoot, and whether they thought it was relevant in treating clubfoot providing a reason for that. In addition, participants were also asked if they had previous experience or exposure to other methods of managing clubfoot and whether they felt the PM was better than other mentioned methods. Participants were also asked to rate their satisfaction of the PM and provide explanations for their opinions. Section C of the questionnaire consisted of a five point Likert scale from strongly disagree to strongly agree, inquiring about the perceived barriers and challenges faced by the medical rehabilitation professionals when delivering treatment of clubfoot using the PM. The participants were also asked to provide possible recommendations for improving the effectiveness of the PM in their clinical setting.

In a preliminary study, the questionnaire was tested for content validity by four experienced physiotherapists and one occupational therapists trained in the PM working as lecturers at the University of Zimbabwe, College of Health Sciences yielding a scale-level content validity index (S-CVI) of 0.71. After the validation, a test retest reliability of the English version of the questionnaire was conducted using 10 medical rehabilitation professionals working at a provincial hospital 80 km from Harare who had previously received formal training on the method and were using the technique in their daily practice. The re-test was conducted seven days later. The kappa coefficients of the primary ordinal outcome measures ranged from 0.32 to 0.72.

### Procedure

The study was reviewed and approved by the Joint Research Ethics Committee for the University of Zimbabwe, College of Health Sciences and Parirenyatwa Group of Hospital (JREC; ref number = 266/14) and Medical Research Council of Zimbabwe (MRCZ; reference number = B/774). In addition, permission to involve medical rehabilitation professionals was obtained from their regulatory council called the Medical Rehabilitation Practitioners Council of Zimbabwe (MRPCZ). Clinical directors of all the three public hospital gave written permission for the study to be conducted. Written informed consents were obtained from medical rehabilitation practitioners who volunteered to participate in the study.

### Data collection

After getting ethical and institutional approvals, data collection was conducted between January and March 2015. Data collection was divided into two stages: preparatory stage and questionnaire administration. In the preparatory stage, the researchers visited the research settings consecutively to explain the procedural issues of the research to the participants, to give them information letters and making appointments for data collection with them. This was necessary to minimise interrupting treatment sessions for patients. Thereafter, data collection was conducted for a period of one month, on dates agreed upon with the participants. The questionnaires were self-administered to the participants during their spare time and were collected immediately upon completion. The researcher was available to answer any question the participants had regarding the questions. This was done as well to minimise practitioner to practitioner discussions about the questions and responses.

### Data analysis

Statistical analysis was performed using STATISTICA version 11. All responses from open ended questions were grouped into categories and equated to frequencies. Descriptive statistics such as the mean with the standard deviation were used to describe normally distributed continuous data and the median with the interquartile range was used to describe skewed data. The Shapiro Wilk test was used to assess for normality of age data. Data generated from open-ended questions were coded into categories for further analysis and then a thematic analysis was done.

## Results

### Baseline characteristics of the participants

Table 1 shows the sample characteristics for the participants. The sample had 27 (65.9 %) females and 14 (34.1 %) males. The median age of the participants was 36 years (interquartile range, 24–42 years). Of the 41 participants, there were 27 (65.9 %) physiotherapists, 11 (26.8 %) rehabilitation technicians and 3 (7.3 %) occupational therapists. The majority of the medical rehabilitation professionals included in the study were working in the government institutions (35, 85.4 %) and 6 (14.6 %) were private practitioners. The majority of the participants (29, 70.7 %) had received formal training through ZSCP sponsored workshops, whereas 12 (29.3 %) were trained during their undergraduate degree at the University of Zimbabwe in fulfilment of their physiotherapy and occupational degree programmes. The majority of the participants (31, 75.6 %) were senior practitioners with more than five years of clinical experience. The mean number of children treated for clubfoot with the PM by each participant per week was 8 with a standard deviation of 1.96. Uncomplicated congenital talipes equinovarus (CTEV) was the most common type of clubfoot treated using the PM in the clinical settings included in the study (38, 92.7 %).

**Table 1:** Baseline characteristics of the participants (*n* = 41)

Characteristic	Number	Percentage
Gender		
Male	14	34.1
Female	27	65.9
Profession		
Physiotherapy	27	65.9
Occupational Therapy	3	7.3
Rehabilitation Technician	11	26.8
Institution of work		
Public hospitals	33	80.5
Private Clinics	6	14.6
University	2	4.9
Years of experience		
<5 years	10	24.4
≥5 years	31	75.6

### Perceptions towards Ponseti method of treating clubfoot

All participants indicated that they were actively involved in the weekly clubfoot clinics at their respective hospitals and clinics at the time of the study. However, 26 (63.4 %) had used other previous methods such as the Kite method in the correction of the clubfoot deformity prior to the training by the Zimbabwe Sustainable Clubfoot Programme. Their experience with the Kite method was on average of 6 years (SD = 2.86). The other rehabilitation professionals had no previous practical experience with any method of treatment.

**Fig. 1:**
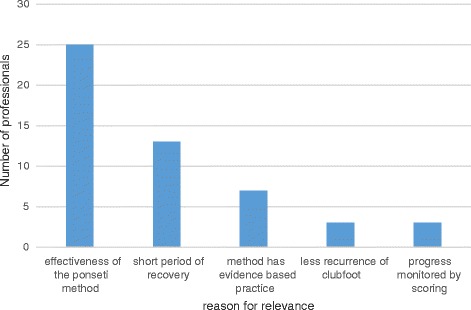
Reasons given by the participants on the relevance of the Ponseti method

**Fig. 2:**
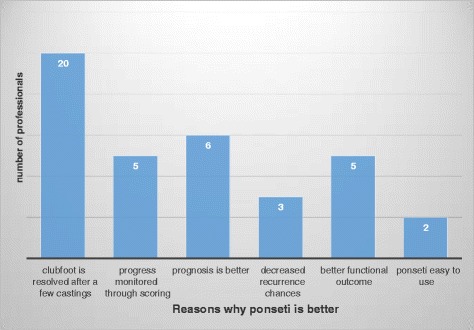
Reasons why the Ponseti method is preferred to other methods used previously

Responding to the question about the relevance of the Ponseti technique, all participants felt that the method was relevant. Figure 1 shows some of the reasons described by the participants for the relevance of the PM. When asked to express their satisfaction with the results of the PM, all participants agreed that it yields better clinical outcomes or results than previous methods. Figure 2 shows some of the reasons given verbatim for the satisfaction with the PM. The majority of the participants felt that if the technique is correctly applied the clubfoot resolves early with only few castings.

### Perceived challenges in using the Ponseti method

The participants were asked to indicate preselected list of barriers and challenges they are likely to encounter in their clinical work when treating clubfoot using PM on a Likert scale from strongly agree to strongly disagree. At the end of that section in the questionnaire, they were also asked to give their free perceived challenges using open-ended questions. One major challenge highlighted by the participants was the issue of compliance. The majority (25, 61.0 %) agreed or strongly agreed that non-compliance to treatment is a major issue of concern. In addition, 22 (53.7 %) of the participants agreed or strongly agreed that caregivers of children with clubfoot lacked insight on the Ponseti technique. Twenty (48.8 %) of the participants had a neutral perception on whether religion of the caregivers was a barrier to treatment, whereas 14 (34.1 %) agreed or strongly agreed that religion had a negative impact on treatment.

On the issue of the adequacy of their training, the majority of the participants 27 (65.9 %) agreed or strongly agreed that it was adequate. Other indicated challenges included lack of resources at the clubfoot clinics, mentioned by 23 (56.1 %), of the participants, and increased workload for the clinicians, agreed by 18, 43.9 %, of the participants.

### Recommendations for improving clubfoot management

Out of the 41 participants in the study, 25 (61.0 %) provided recommendations for improving clubfoot management using the PM by answering the respective open ended question. The responses have been thematically summarised and are shown in Table 2. The participants felt that there is need to decentralise the services from tertiary health institutions to the community or district health facilities for easy access of the majority of the population. Some health professionals reported highlighted the need for more training, not only on the clinical application of the PM but also on the handling of children with the clubfoot, especially those with multiple disabilities.

**Table 2:** Recommendations for improving the Ponseti method (*n* = 25)

Recommendation	Number (%)
There is need for decentralization of services to provincial and district hospitals and clinics.	8 (32)
There is need for more outreach programmes and awareness campaigns for the community on clubfoot.	1 (4)
There is need to ensure that one medical rehabilitation practitioner attend to one child with clubfoot throughout the entire treatment process.	2 (8)
There is need for caregivers’ training on child handling undergoing treatment with the Ponseti method.	5 (20)
There is need to increase the number workshops for the continued training of rehabilitation professionals.	3 (12)
There is need for Plaster of Paris (POP) to be provided by the respective hospitals instead of caregivers buying the material.	1 (4)
There is need for the change of brace type given to children.	2 (8)
There is need for support visits to other hospitals for capacity building and training.	I1 (4)
There is need for high beds to work on to prevent back pain.	2 (8)

## Discussion

Clubfoot is a common problem in Zimbabwe with minimum incidence rates comparable to the rest of the world [9]. With the recent introduction of the PM in the country as the preferred method of treatment of clubfoot and the continued investment by the ZSCP in the training of health workers across the country, there is a huge need to evaluate the effectiveness of the PM in managing clubfoot. Our study, which is the first in the country, makes such an attempt by documenting the opinions of medical rehabilitation professionals, who are the service providers, on the application and effectiveness of the PM for clubfoot management. This issue has been rarely investigated, so comparisons with other studies are difficult. This study should be seen a pilot study just describing baseline opinions of a sample of trained medical rehabilitation professionals largely working in public hospitals in Harare, Zimbabwe.

The results of the present study may be important for generating a hypothesis on the perceptions of effectiveness of PM as it is applied in Harare hospitals to correct the clubfoot deformity. The perceived opinions of the participants enrolled in the study, however, might not correlate with the actual results of treatment. This is a major limitation of this study. Future studies using larger samples and robust study designs are needed to fully understand the effectiveness of the Ponseti method in correcting the clubfoot deformity in Zimbabwean hospitals.

The present study targeted medical rehabilitation professionals because all the established clubfoot clinics in large central hospitals in Harare are run mainly by physiotherapists in collaboration with occupational therapists and rehabilitation technicians. The central role of paramedical health care professionals in the management of the clubfoot has been consistently reported in literature. In Uganda, paramedical healthcare professionals run clubfoot clinics in large hospitals [20]. In Malawi, orthopaedic clinical officers are heavily involved [24]. In South Africa, physiotherapists are also heavily involved [23]. In Zimbabwe, physiotherapists, occupational therapists and rehabilitation technicians work together during the clubfoot clinics. This is primarily due to resource allocation. At any hospital in the country, all these professionals work in the same department. In addition, the clubfoot clinics are run on weekly basis at every hospital in the country, hence collaboration is inevitable. This organisation ensures adequate human resources for the clinics, which are normally replete with patients. The local hospital doctors only come in to perform the tenotomies, when necessary. On the other hand, rehabilitation technicians, by nature of their training which provide them with some competences in physiotherapy, occupational therapy and speech therapy, attend to all patients with musculoskeletal conditions. This probably explains the increased number of rehabilitation technicians in the present study compared to occupational therapists.

In terms of demographics, the final sample of participants had more females than males, a true reflection of the gender distribution of medical rehabilitation professionals in Zimbabwe. Most participants trained in the PM were working in government health institutions rather than private rehabilitation clinics. This relates directly to the fact that the ZSCP sponsored clubfoot clinics were established in large government referral hospitals to localise the services and involve the human resources available in these settings. The majority of the participants were senior clinicians with five or more years of experience in clinical settings. Their opinion and general impression on the PM application and its relative effectiveness in this setting are important to consider given that they had probably witnessed other methods of correcting the clubfoot before the PM was introduced. Indeed, most of the participants stated that they had previous exposure and practical experience with the Kite method.

### The effectiveness of the Ponseti method

All medical rehabilitation professionals felt that the PM is relevant and effective in treating clubfoot in children. These findings are consistent with a number of studies that have reported excellent results with the PM [7, 15, 22–27]. The PM has reduced total health care costs, clubfoot surgery frequency and has also changed the patterns of surgery performed for clubfoot in Nigeria [25]. Similar findings were reported in India [15]. Interestingly, participants in the present study who had prior experience in other conservative methods such as the Kite method felt that the PM was more effective. The opinions and impressions of the clinicians confirm that the PM should be considered a standard treatment of congenital talipes equino varus. Shabtai et al. [28] reported that PM has become the gold standard for the treatment of idiopathic clubfoot over the last two decades. In the present study, idiopathic CTEV was the most common type of clubfoot diagnosed in the children. On average, the participants were attending to eight children every week with this condition. This indicates the frequency of use of the PM by the medical rehabilitation practitioners.

A number of reasons were postulated for the relevance of the PM compared to other previously used methods. The PM was perceived to be effective yielding better clinical results improving the functional prognosis of the condition in a shorter period. This is consistent with other studies [15, 29, 30]. It is suggested that with good technique the clubfoot should be corrected in two months with weekly castings [17]. In addition, the participants felt that the Ponseti technique was understandable from a biomechanical, anatomical and scientific point of view unlike the previous methods. Abbas et al. [27] attributed 95 % success rate in correcting clubfoot in clinical practice to sound understanding of the patho-anatomy of clubfoot. Consistent with these findings, Ponseti [17] recommends sound understanding of the functional anatomy in the manipulation and casting of the foot for an effective correction of the deformity. Methodical and meticulous application of the casts and braces possibly minimize the risk of recurrences. Recurrences, however, are known to occur in up to one-third of patients in spite of 100 % success rate which may be achieved with the initial correction [31]. Nevertheless, in the present study, the participants highlighted the potential of less recurrences with the PM as another important reason for the relevance of the Ponseti technique as compared to previous methods. These findings are consistent with other results reported in the literature [21].

### Challenges faced in the treatment of clubfoot using Ponseti

Among the perceived barriers and challenges mentioned by the participants, the most outstanding was the lack of adequate insight of the PM by the caregivers. Although more research is needed on this issue, these findings suggest a need to educate the caregivers of children with clubfoot on the PM and the implications for defaulting treatment. Non-compliance to treatment was also highlighted as a challenge faced by the clinicians. Future studies are needed to determine the level of knowledge of the treatment program and expectations among caregivers and assess for an association with defaulting history. The risk associated with defaulting weekly treatments should be probably be emphasised to caregivers to increase their awareness. Because of the long term rehabilitation required for the clubfoot, non-adherence to hospital appointments is inevitable. However, it should be addressed and impressed on the caregivers from the outset. Rigid adherence to the clubfoot programme has been reported to be associated with decrease in the rate of relapse and number of patients requiring more extensive surgical intervention [29].

In Uganda, in order to address the challenge of lack of compliance to treatment of clubfoot, posters and pamphlets were designed and distributed to village health teams, healthcare centers, churches, and schools [20]. They also employed radio as a way to educate the population at large [20]. Although much more community education is still required, these efforts are commendable and provide an effective platform from which to launch further programs and efforts. In Peru, in a study evaluating the barriers to using the PM among physicians, 27 of 32 (84.4 %) felt that the lack of parental knowledge about the PM was a barrier to its success [5]. Inadequacy of knowledge, non-compliance of caregivers and financial constraints were also highlighted by number of other studies in the literature [15, 30, 32]. Lack of resources to use during treatment was also indicated strongly as a barrier to effective treatment by 23 (56.1 %) of the participants in the present study. These findings are consistent with a number of studies [30, 33, 34]. In the present study, increased patient demand for treatment at clubfoot clinics could account for the lack of adequate resources in the local hospitals. This highlights the huge need for more rehabilitation professionals trained in the PM.

### Recommendations for improving clubfoot management

Recommendations and suggestions on how to improve the PM in Zimbabwe were given based on the current situation, challenges and previous experiences of the medical rehabilitation professionals in the treatment of the clubfoot deformity. The outstanding recommendation was that the services must be decentralised to the smaller health centres so as to reduce the workload in the central hospitals. It is possible that as the PM gains credence and increase access for rural patients, there will be increasing demand for the services in tertiary institutions. In Uganda, clubfoot detection and treatment is being decentralised to improve access to the facilities [20]. Since 2011, the Zimbabwe Sustainable Clubfoot Programme has been conducting training in-service training workshops countrywide in a bid to decentralise the service to grassroots level.

It has been suggested that caregivers need to be educated about the details pertaining to their child’s treatment and outcome expectations so that they see the importance of compliance throughout the course of treatment and maintenance [20]. The same suggestion has been given in this study by the medical rehabilitation professionals that caregiver understanding of clubfoot deformity and the PM can improve compliance with all stages of treatment. Increasing level of awareness could drastically improve the rate and spread of success of the Ponseti method and reduce the risk of recurrences.

## Conclusions

The study’s aim was to determine the impressions and opinions of medical rehabilitation practitioners on the method in terms of its effectiveness, perceived challenges and possible recommendations for improvements on the use of the PM in treating clubfoot in Harare hospitals. The study revealed that the PM is being applied as treatment of choice in the management of clubfoot by the medical rehabilitation professionals in this setting. The PM has been described as the most relevant method for treating children with clubfoot, yielding better clinical outcomes than previously used methods. The positive feedback from the care providers about the PM effectiveness along with their recommendations of decentralising the services for easy access by children in rural areas highlights the need for more rehabilitation professionals trained in the method.
